# Oncoplastic breast-conserving surgery using a modified Grisotti’s flap for a patient with central breast cancer in a non-ptotic breast

**DOI:** 10.1007/s00595-025-03047-5

**Published:** 2025-04-25

**Authors:** Yuko Kijima, Munetsugu Hirata, Yumika Nakazawa, Kazuya Shinmura, Naoki Hayashi, Ryunosuke Kijima, Hisamitsu Zaha

**Affiliations:** 1 Department of Breast Surgery, Fujita Health University, School of Medicine, 1-98 Dengakugakubo, Kutsukake-Cho, Toyoake, Aichi 470-1192 Japan; 2 Nakagami Hospital, 610 Noborikawa, Okinawa, Okinawa 904-2192 Japan

**Keywords:** Breast cancer, Oncoplastic breast surgery, Grisotti’s flap

## Abstract

The treatment of patients with early breast cancer using breast-conserving surgery (BCS) commonly leads to local control and acceptable cosmetic results. We herein report a useful technique to achieve symmetry of the breast shape and size for a central lesion of the breast. A Japanese patient with early breast cancer located in the central area of the breast was enrolled in this study. Intraductal spread to the nipple was suspected. We resected the cylindrically shaped breast tissue with the nipple–areola complex and repaired the resected area using a modified Grisotti technique, a volume displacement technique using breast tissue with extra-breast tissue. The modified Grisotti flap technique during oncoplastic breast-conserving surgery (OPBCS) may thus be useful for patients who desire a symmetrical breast shape and size.

## Introduction

Breast-conserving surgery (BCS) is a well-established treatment for breast cancer that facilitates local disease control with acceptable cosmetic results [1, 2]. After locoregional control and survival, a good cosmetic outcome is one of the main aims of BCS [3, 4]. Resection of the nipple-areola complex with the central area of the breast and simple closure of the defect, both vertically and horizontally, gives the breast a particular shape [5].

We previously reported oncoplastic techniques for three patients with breast cancer located in the central area of the breast: one case of nipple–areolar conservation, one of free nipple–areolar grafting, and one of nipple–areolar resection [6]. We successfully performed oncoplastic breast-conserving surgery (OPBCS) using volume displacement involving a modified Grisotti flap in a Japanese patient with non-ptotic, moderately sized breasts. We herein report an OPBCS technique that combines volume displacement of the breast and extra-breast tissue.

## Patient

A 51-year-old Japanese patient diagnosed with early stage breast cancer was enrolled in this study. The lesion was diagnosed as ductal carcinoma in situ and spread toward the nipple. Preoperative MRI and ultrasonography revealed intraductal spread to the nipple.

The patient desired breast conservation, not total mastectomy, followed by immediate breast reconstruction. The patient provided informed consent for OPBC, involving volume displacement using a modified Grisotti flap. The indications for OPBCS using the modified Grisotti flap were as follows: (1) the cancer lesion was restricted to the central area of the non-ptotic breast; (2) ductal spread to the nipple-areola complex was detected; (3) informed consent was obtained preoperatively after an explanation of the surgical procedure; and (4) she was not concerned about the length of the surgical scar. She was also informed about the operative plan and given different surgical options: other oncoplastic surgical techniques, such as a free nipple–areolar grafting technique, areola-preserving surgery, or OPBCS with nipple reconstruction using a spiral-peeling technique [9]. Finally, we proposed volume displacement using a modified Grisotti flap, which combined OPBCS, volume displacement using both intra- and extra-mammary tissues, and reconstruction of a new inframammary line (IML). Additional informed consent regarding the risk of skin–glandular flap necrosis and the possibility of remaining cancerous lesions was obtained.

## Surgical procedure

### Design

**Fig. 1:**
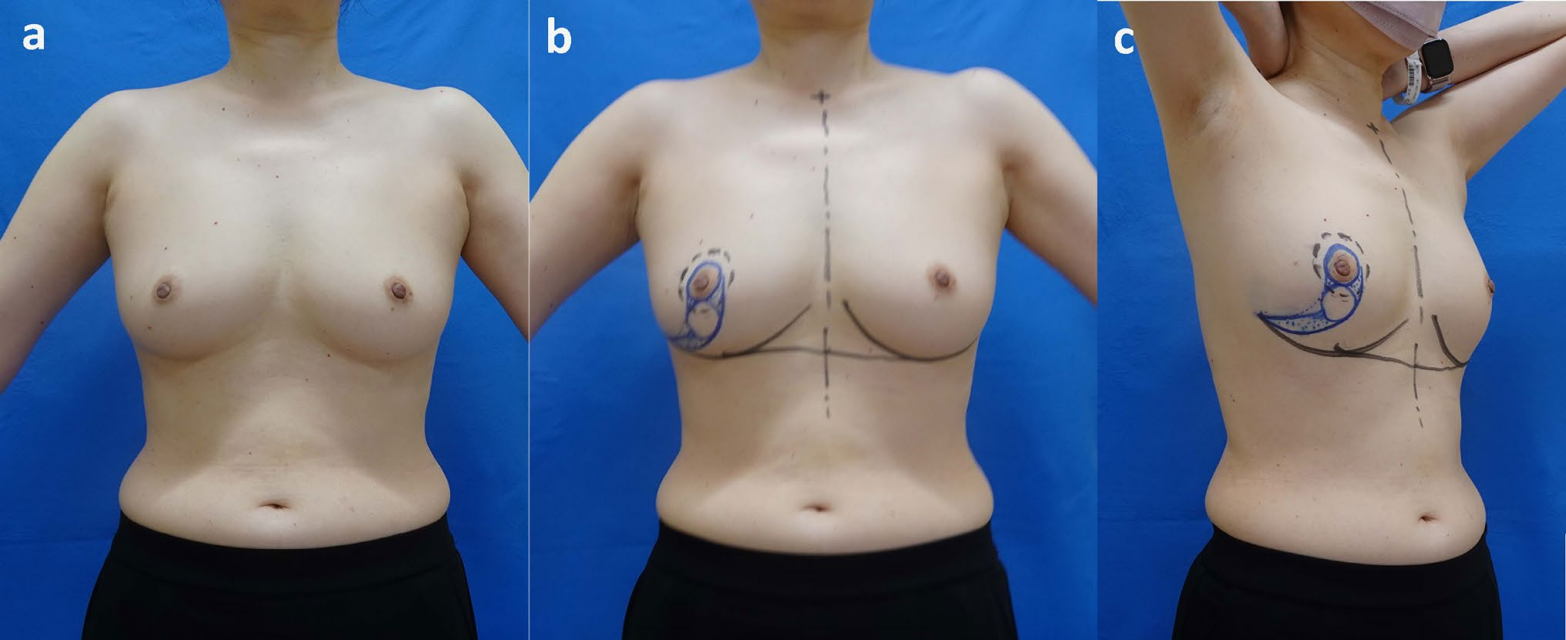
Preoperative findings. **a** Preoperative findings. **b**, **c** Design of OPBCS

**Fig. 2:**
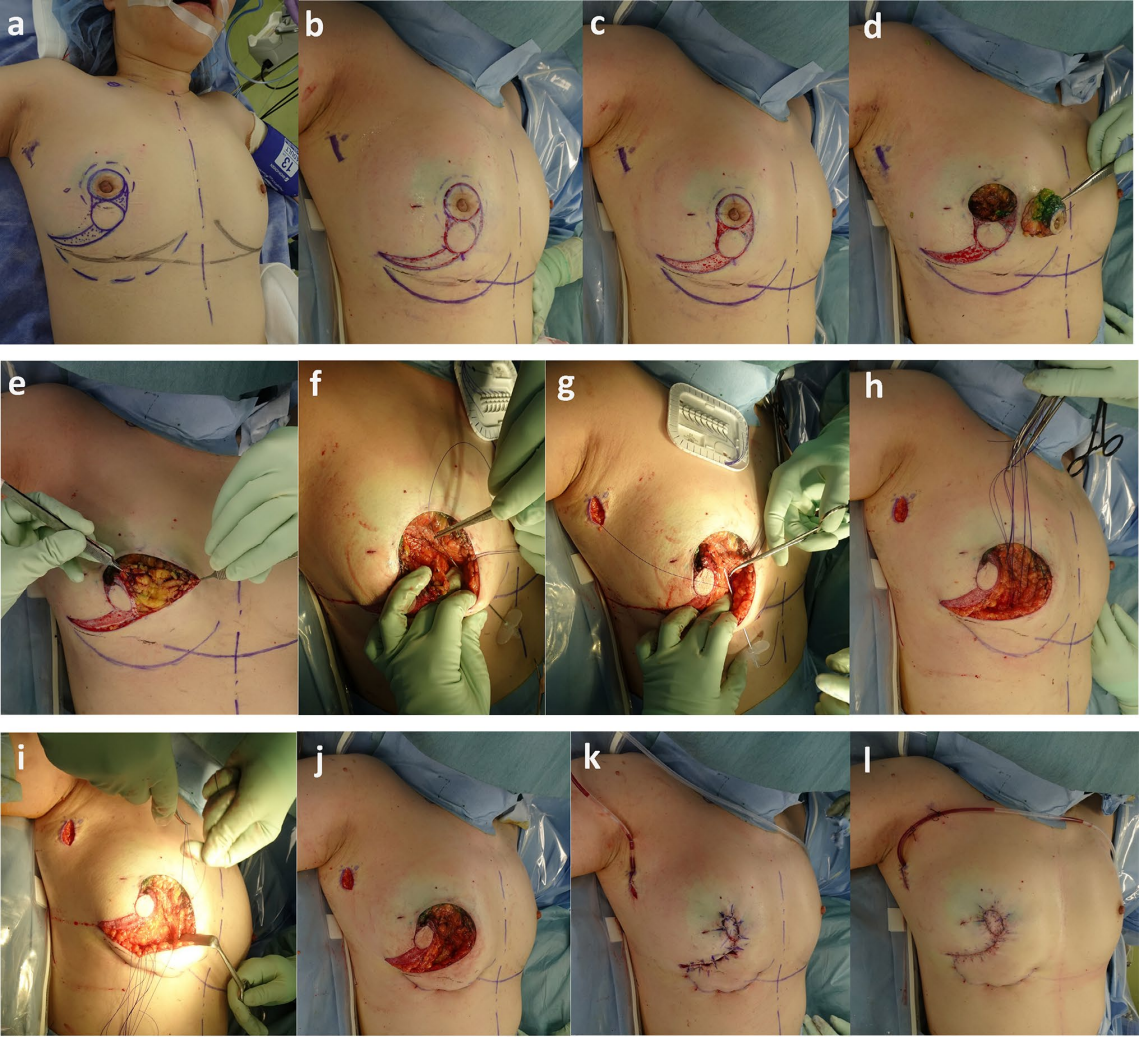
Procedure of OPBCS using a modified Grisotti’s flap. **a** Two J-shaped lines were drawn from the edges of the areola at 9:00 and 3:00. In this field, a circle was drawn to fill the cavity after resection of the nipple–areola complex. A caudal curve 20 mm from the IML is drawn (purple dotted line). **b** All curves were initially cut until the superficial layer of the skin. **c** The dotted area was de-epithelialized. **d** Columnar-shaped breast tissue with the nipple–areola complex was resected. **e**–**f** The 2–0 PDS® II dermis and epidermis were placed using a 16 G needle along the curve below the IML. **h** Five 2–0 PDS® II sutures were placed on the new IML and tied toward the cranial side. **j** Each 2–0 PDS® II was tied without attaching to the thoracic tissue. **k**. Several stitches using absorbable sutures were added between the skin–glandular flap and the remnant gland. **l** Findings at the end of the operation

She was referred by a breast surgeon (Y.K.) before surgery at the outpatient clinic and provided her informed consent. The day before surgery, we designed and created a drawing based on the original Grisotti technique. The surgical margins of the skin were drawn on the areola. Two J-shaped curves were drawn from the edge of this circle surrounding the areola toward the lateral-caudal edge of the breast. One circle was drawn between the two J-shaped lines (Figs. 1, 2a and b). The surgical margins of the breast tissue were drawn in dotted-black ink surrounding the areola (Fig. 2b, c). In the standing position, the IML levels were symmetrical (Fig. 2b, c).

### Sentinel lymph node biopsy

A sentinel lymph node biopsy (SNB) using the RI and dye method was performed, which required another incision in the axillary area. One sentinel lymph node was biopsied and histologically examined during the surgery. No metastatic lesions were observed.

### Oncoplastic breast surgery

**Fig. 3:**
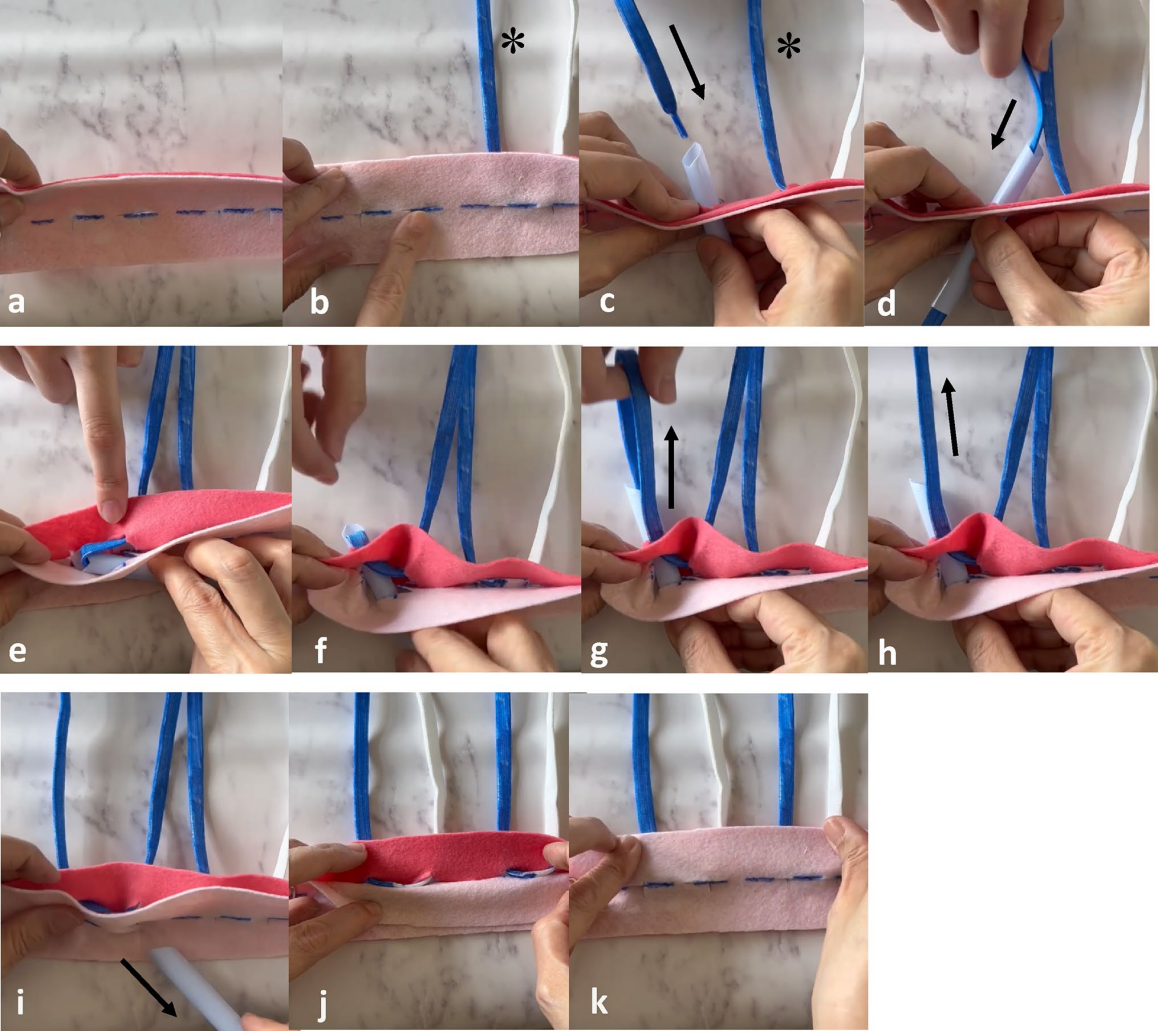
The model of the creation of a new inframammary line (IML). **a** The blue wavy lines indicate the position where the new IML should be. **b** One suture was already made into the dermis (*). **c**, **d** Light pink represents the epidermis, whereas dark pink represents the dermis. A light blue straw, representing the needle, was inserted perpendicularly from the epidermis into the subcutaneous tissue. The absorbable suture was passed from the needle surface through the needle hole, guiding the suture tip toward the epidermal side. **a** With the suture in place, the needle tip was withdrawn to the layer between the epidermis and dermis, where it was then rotated 90°and slid by approximately 10 mm within this layer. **b** The needle tip was again rotated 90°and penetrated the dermis while keeping the suture in place. **g**, **h** We extracted the suture from the needle lumen. **i** The needle was removed while the suture remained. **j** The suture was adjusted so that its center aligned with the center of the puncture site. **k** The suture was completely hidden beneath the skin surface

**Fig. 4:**
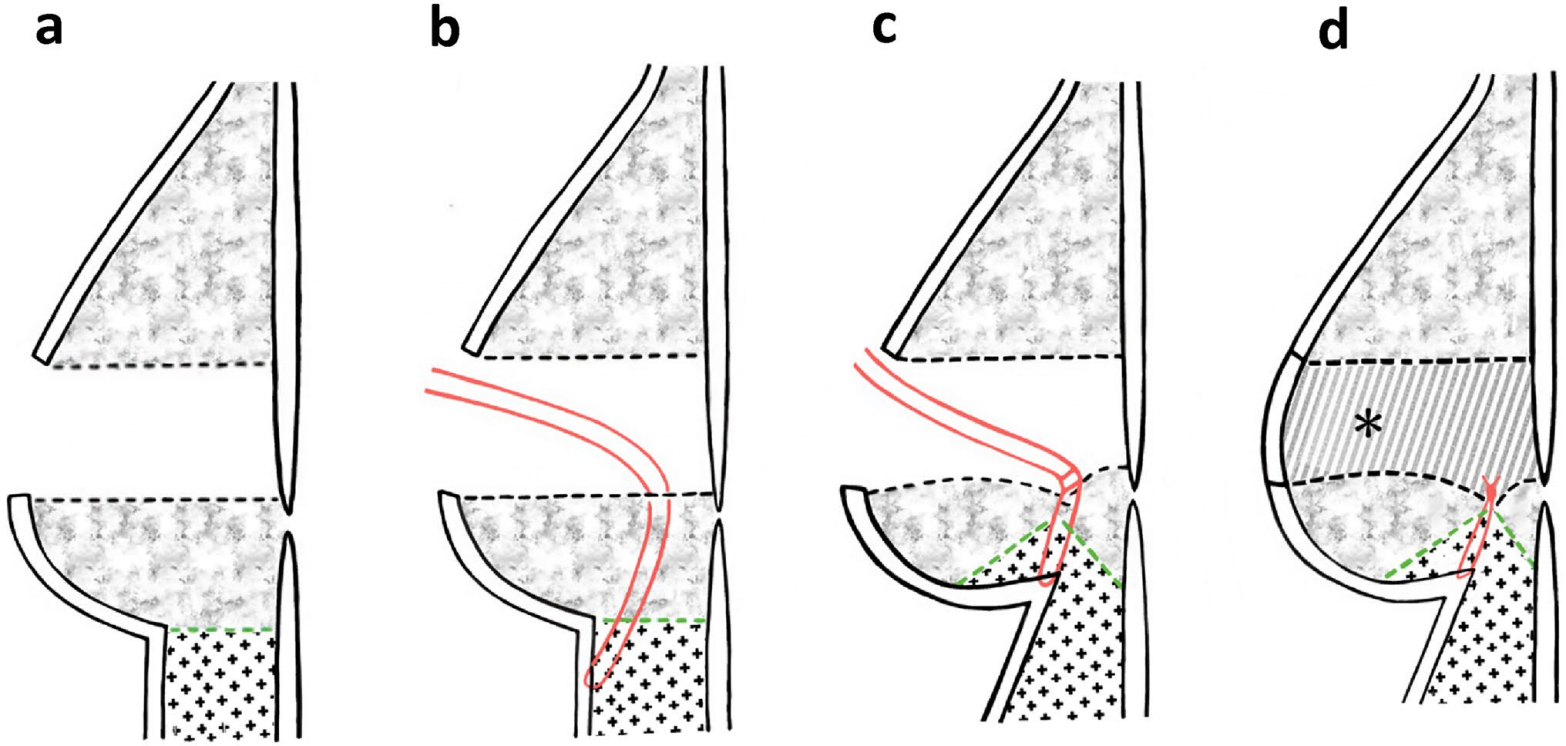
**a** The central area of the breast was resected with the nipple areola. **b** The suture was placed between the epidermis and dermis at the location of the new IML. **c** We tied the suture while traction was applied to the skin and tissue on the inferior side of the breast. It is important to adjust the degree of suturing to ensure that the new IML is positioned symmetrically with the opposite breast. d. The central defect was displaced by a skin–glandular flap (*)

First, we drew a caudal curve 20 mm from the IML (Fig. 2a; blue dotted line). Two J-shaped lines were drawn from the edges of the areola at 9:00 and 3:00. In this field, a circle was drawn to fill the cavity after resection of the nipple-areola complex (Fig. 2b). The dotted area in the J-shaped band was completely de-epithelialized (Fig. 2c). The cancerous area was resected cylindrically along with the nipple-areola complex (Fig. 2d). The inner line of the J-shaped area was cut from the surface of the dermis to the bottom of breast tissue (Fig. 2e). The width of the J-shaped band was narrowed toward the caudal side to be sutured to each other. The circle-skin and breast tissue under the de-epithelialized area were displaced toward the cranial side to fill the partial defect at the center of the breast. We placed a 2–0 synthetic absorbable monofilament suture (2–0 PDS® II (polydioxanone) suture (Ethicon, Inc., Cincinnati, OH, USA) in the dermis and epidermis using a 16 G needle along the curve 20 mm caudal to the IML. Five 2–0 PDS® II were placed and tied toward the cranial side (Fig. 2f-i). We describe this surgical technique in Fig. 3 using our model. The blue wavy lines indicate the position of the new IML. Light pink represents the epidermis, whereas dark pink represents the dermis (Fig. 3c-k). One suture is placed in the dermis (*). The top half is colored blue, while the tail half is white. A light blue straw, representing the needle, was inserted perpendicularly from the epidermis into the subcutaneous tissue. The absorbable suture was passed from the needle surface through the needle hole, guiding the suture tip toward the epidermal side (Fig. 3c, d). With the suture in place, the needle tip was withdrawn to the layer between the epidermis and dermis, where it was then rotated 90°and slid by approximately 10 mm within this layer (Fig. 3e). The needle tip was again rotated 90°and penetrated the dermis while keeping the suture in place (Fig. 3f). After extracting the suture from the needle lumen, the needle was removed (Fig. 3g-i). The suture was adjusted such that its center was aligned with the center of the puncture site (Fig. 3j). The suture was completely hidden beneath the skin surface (Fig. 3k). The suture was tied, while traction was applied to the skin and tissue on the inferior side of the breast. It is important to adjust the degree of suturing to ensure that the new IML is positioned symmetrically with the opposite breast (Fig. 4a-c). This procedure allows the skin and tissue below the inframammary fold to be repositioned into the breast, while maintaining a clear inframammary fold angle.

After the creation of the new IML, the skin–glandular flap composed of de-epithelialized skin, circulated skin, and breast tissue was rotated to the defect. Several absorbable sutures were added between the skin–glandular flap and the remnant gland. The central skin defect was completely repaired, involving not only the volume, but also the central area of the skin (Fig. 2k, Fig. 4d). The final findings are shown in Fig. 2l.

### Pathological findings

The pathological diagnosis was noninvasive ductal carcinoma of the breast. All surgical edges were cancer free. The patient underwent irradiation of the remnant breast tissue.

## Results

**Fig. 5:**
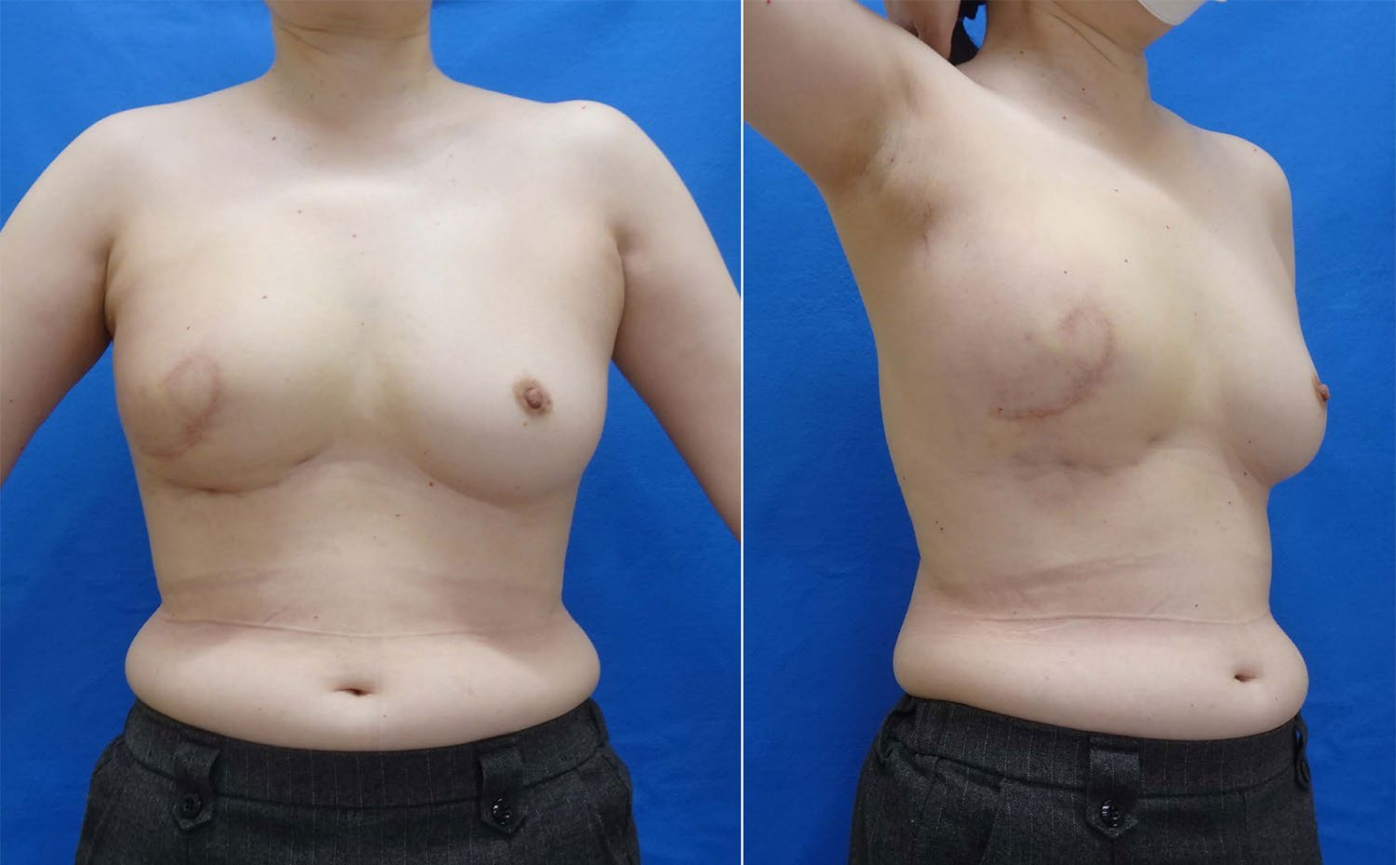
Postoperative findings at 1 year

There were no postoperative complications such as bleeding, infection, fat necrosis, or blood flow disorder of the reconstructed breast. Excellent symmetry regarding both the size and shape of the breast was achieved, including the IML level (Fig. 5). Neither distant nor local recurrence was noted over the follow-up period of 18 months postoperatively.

## Discussion

According to the literature, breast cancer located in the central area of the breast accounts for 5 to 20% of all breast cancer cases [10, 11]. When performing traditional BCS for central breast tumors, NAC and the tumor are excised en bloc, and the defect is closed using either a wedge excision or a purse-string suture. However, many women are dissatisfied with the cosmetic results [12, 13]. In 1993, Galimberti et al. reported an oncoplastic procedure involving a Grisotti flap for the management of centrally located breast cancer. They described the technique as an excision of NAC directly over the site of the tumor, extending down to mobilize a dermoglandular flap, which is then de-epithelialized to reshape the breast cancer and recreate the areola [14]. We introduced displacement using Grisotti’s flap for centrally located breast cancer in Japanese patients and found that this simple technique generated excellent results [15]. Until this study, we considered that the indications for volume displacement using Grisotti’s flap were limited to patients with middle-sized or large and/or ptotic breasts, and the skin was soft. However, we reported several experiences of OPBSC for patients with small- to middle-sized breasts; the volume replacement technique with extra-mammary tissue was adequate to fill the defect and thereby obtain symmetrical results [16–20]

Based on these experiences, Grisotti’s flap may be useful for the repair of central breast defects in patients with relatively large breasts. We considered that there was a limitation of Grisotti’s technique for patients without large breasts; a higher excision ratio to the breast leads to worsening cosmetic results, especially in patients with small-to medium-sized breasts. Conversely, we noticed that volume displacement using Grisotti’s flap was an excellent technique for repairing central breast defects. In addition, we encountered two patients requiring OPBCS. One involved modifying V-mammoplasty for a cancer lesion in the inner lower area of a large breast in the same way as for a patient with a non-ptotic small breast [21]. The other involves creating a new IML and elevating the tissue below the true inframammary area to form part of a new breast mound [22]. Based on this experience, in the present case, we modified the original flap by adding skin and fatty tissue to the extra-breast area in a similar manner. If we performed Grisotti’s technique in this patient without a non-ptotic, middle-sized breast, the size of the operated breast would be smaller than that of the contralateral breast due to loss of the skin and breast tissue. The IML of the treated breast must have been moved to the cranial side. It is also predicted that it would be difficult to reproduce the angle of the inframammary area, which we achieved by using the modified Grisotti’s flap, volume displacement, volume replacement, and reproducing the IML.

In this study, the Japanese patient had a medium-sized and non-ptotic breast. We planned to resect the central area of the breast with the nipple–areola complex and reconstruct the breast mound using the modified Grisotti flap. We resected 29 g of tissue; the total operating period was 89 min, and reconstruction took 53 min. No complications were noted either during or after the surgery. Partial defects were repaired using skin fatty tissue in the lateral area of the breast. We also repaired a central circular defect of the areola. The remaining breast volume in the inner to lower area of the breast was increased to equate with the original breast tissue using caudal skin fatty tissue.

This resulted in an adequate volume to fill the defect and make the breast symmetrical. Therefore, it is important to avoid drawing a new IML that is extremely low. The appropriate distance between the new and original inframammary fold lines is unclear. However, based on our past experiences, a distance of approximately 15–20 mm may be reasonable for patients without breast ptosis and relatively small breasts, for whom this technique may be useful.

In this study, the inner line of the J-shaped area was cut from the surface of the dermis to the bottom of breast tissue (Fig. 2e). Cutting the outer line instead and attaching Grisotti's flap caudally might allow the flap, along with the inframammary fold tissue, to be more efficiently moved cranially. Further modifications such as this would make Grisotti’s technique more useful, although it involves a significant alteration of the procedure.

A contraindication for this technique is the use of a large areola for a relatively small or fatty breast, similar to Grisotti’s methods.

When repairing a central breast defect using Grisotti’s flap or a modified Grisotti’s flap in patients with ptotic breasts, it may be difficult to maintain symmetry because of the size of the original breasts.
